# Endovascular treatment for ureterorectal-arterial fistula and external iliac artery stenosis caused by recurrent endometrial cancer invasion: A case report

**DOI:** 10.1016/j.radcr.2025.05.039

**Published:** 2025-06-13

**Authors:** Masaki Yoshikawa, Shohei Chatani, Kenta Tanimura, Masayuki Shimomura, Satoshi Ishimoto, Yugo Imai, Akitoshi Inoue, Yuki Tomozawa, Yoko Murakami, Akinaga Sonoda, Yoshiyuki Watanabe

**Affiliations:** Department of Radiology, Shiga University of Medical Science, Seta, Tsukinowa-cho, Otsu, Shiga 520-2192, Japan

**Keywords:** Ureterorectal-arterial fistula, Oncology, Interventional radiology, Internal iliac artery embolization, Stent graft implantation

## Abstract

Owing to improvements in anticancer treatment, patients’ overall survival has been prolonged, and late complications occur more frequently than previously. We successfully managed a case of ureterorectal-arterial fistula (URAF) and external iliac artery (EIA) stenosis due to endometrial cancer recurrence using an endovascular approach, including arterial embolization and angioplasty. The patient was a 48-year-old woman undergoing chemoradiotherapy for recurrent endometrial cancer following surgery and adjuvant chemotherapy. Tumor involvement and its response to treatment led to the development of URAF and EIA stenosis. Catastrophic bleeding occurred from URAF, and hemostasis was successfully achieved by left internal iliac artery embolization using a mixture of N-butyl cyanoacrylate and ethiodized oil. Nine days after embolization, angioplasty was performed using a stent graft implantation for EIA stenosis. Endovascular treatment is considered a crucial approach for managing both URAF and arterial stenosis, which is surgically untreatable.

## Introduction

In the last decades, the prognosis for recurrent endometrial cancer has improved owing to the development of treatment options [1]. Locoregional therapy (ie, surgery and radiotherapy), systemic therapy (ie, chemotherapy, targeted therapy, hormonal therapy, and immunotherapy), and their combination have proven beneficial for selected patients. Despite the improvement in life expectancy, severe complications induced by tumor invasion or its treatments should be noted. Uretero-arterial fistulas (UAF) and arterio-enteric fistulas (AEF) are rare conditions that can occur after surgery and/or chemoradiation therapy. Ureterorectal-arterial fistula (URAF), which represents a combination of both, is extremely rare and can be fatal due to a high risk of massive hemorrhage [2]. External iliac artery (EIA) stenosis resulting from tumor invasion is also a severe complication that can lead to lower limb ischemia and pose a significant risk of future hemorrhage. Here, we present a case of URAF and left EIA stenosis associated with endometrial cancer recurrence, which was previously treated with surgery and chemoradiotherapy. The patient was successfully managed by endovascular treatment.

## Case report

### History

**Fig. 1:**
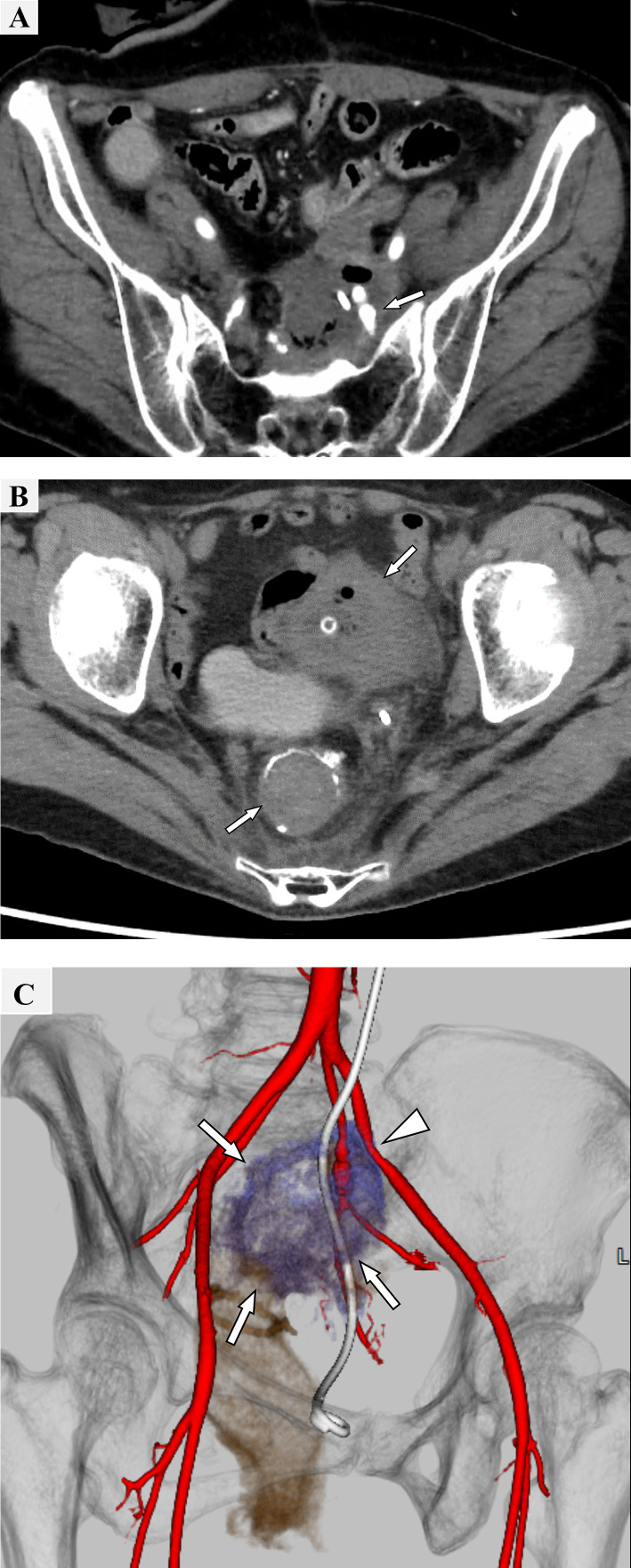
Computed tomography imaging findings of the ureterorectal-arterial fistula. (A) Contrast-enhanced Computed Tomography (CT) in the arterial phase revealed a pseudoaneurysm in the left internal iliac artery (arrow). (B) Noncontrast-enhanced CT revealed a hematoma in the bladder and rectum (arrow). (C) Volume rendering reconstruction revealed that the tumor invaded the left ureter, rectum, and left internal iliac artery (arrow). Pseudoaneurysm formation was detected in left internal iliac artery. Left external iliac artery stenosis was also observed (arrowhead).

A 48-year-old woman was diagnosed with endometrial cancer and presented with rectal invasion and lymph node metastasis. The patient underwent total hysterectomy, bilateral salpingo-oophorectomy, paraaortic lymphadenectomy, low anterior resection, and ileostomy. The invasion of the endometrial carcinoma did not extend to the rectal mucosa, and the patient was pathologically staged as Fédération Internationale de Gynécologie et d'Obstétrique (FIGO) IIIC. Adjuvant chemotherapy (paclitaxel + carboplatin and doxorubicin + cisplatin) and hormonal therapy (medroxyprogesterone acetate) were administered. Two years after surgery, contrast-enhanced computed tomography (CT) revealed localized recurrence in the left pelvic wall. Chemoradiotherapy was performed for the pelvic recurrent lesion, and a double J stent was placed in the left ureter for ureteral stricture caused by tumor invasion. Four years after chemoradiotherapy, the patient presented with severe hematochezia and hematuria and was promptly transferred to our hospital. Upon arrival, the patient was in hemorrhagic shock, with a systolic blood pressure of 80 mmHg and a heart rate of 150 beats/min. The patient’s hemoglobin level was 9.2 g/dL, and because bleeding persisted, the patient received a transfusion of 2 units of erythrocytes. Contrast-enhanced CT revealed a pseudoaneurysm in the left internal iliac artery (IIA), accompanied by a massive hematoma in the rectum and left urinary tract, in which a fistula between the rectum and ureter via the cavity caused by tumor necrosis was suspected (Fig. 1). Furthermore, an arterial caliber irregularity associated with tumor invasion was observed in the left EIA. Due to hemodynamic instability and the presence of pelvic tissue adhesions following previous treatments, surgical intervention was considered inadvisable. Urgent embolization was scheduled for the IIA pseudoaneurysm to stabilize the circulation. Subsequently, elective angioplasty with a stent graft was performed for EIA stenosis was performed to prevent further bleeding complications and lower extremity ischemia, although the patient exhibited no symptoms attributable to the stenosis.

### Embolization for pseudoaneurysm of the left IIA

**Fig. 2:**
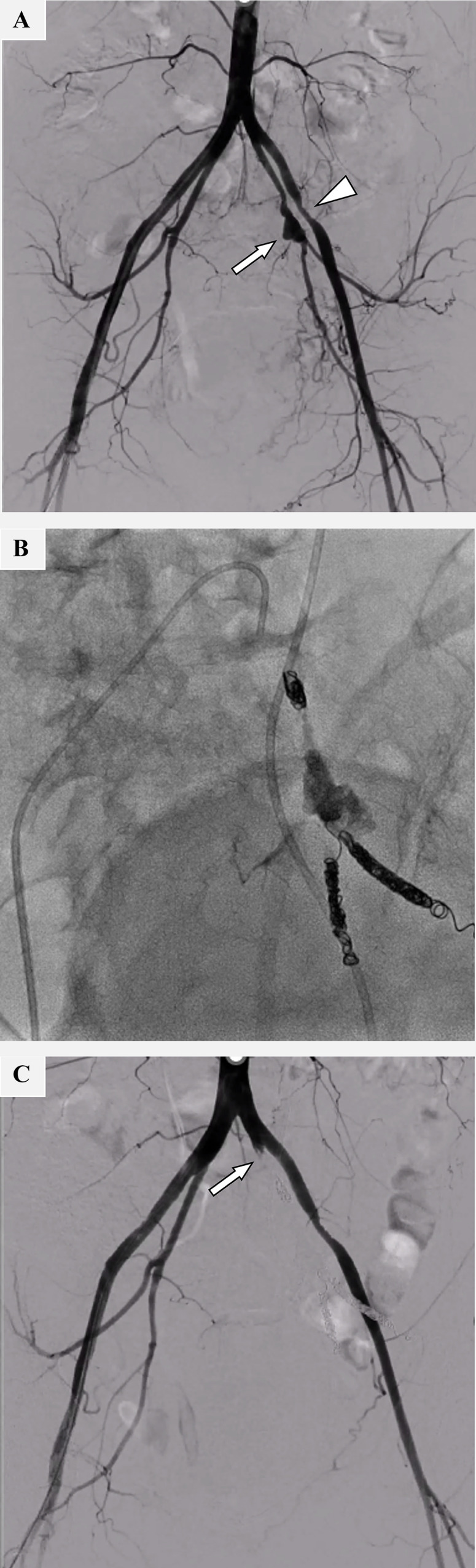
Embolization of the left internal artery pseudoaneurysm. (A) Digital subtraction angiography (DSA) revealed a pseudoaneurysm in the left internal iliac artery (IIA) (arrow). Stenosis in the left EIA was also observed (arrowhead). (B) Embolization of the left IIA pseudoaneurysm. Embolization was performed using several microcoils and mixtures of ethiodized oil and n-butyl cyanoacrylate. (C) DSA after embolization. Complete hemostasis in the left IIA was achieved (arrow).

The embolization procedure was performed through a 5-Fr sheath following a right common femoral arterial puncture. Aortography revealed a pseudoaneurysm in the left IIA and left EIA stenosis (Fig. 2A). A 5-Fr. Cobra-shaped catheter (Hanako, Saitama, Japan) was carried to the left IIA. A 1.9-Fr. microcatheter (Carry Leon, UTM, Aichi, Japan) was advanced over a 0.016-inch guidewire (Meister, ASAHI INTECC, Aichi, Japan). The superior and inferior gluteal arteries were embolized with several microcoils (Tornado, Cook Medical Japan, Tokyo, Japan: C-stopper, Piolax, Kanagawa, Japan), and the pseudoaneurysm was obliterated using a warmed 1:4 (v/v) mixture of n-butyl cyanoacrylate (NBCA) (Histoacryl, B. Braun Melsungen AG, Germany) and ethiodized oil (Lipiodol, Guerbet, Villepinte, France). A detachable microcoil (Target XL, Stryker, Fremont, CA) was used to embolize the proximal segment of the left IIA (Fig. 2B). After the procedure, complete embolization of the left IIA was achieved without any complications (Fig. 2C). Subsequently, the patient’s blood pressure and heart rate were stabilized.

### Stent graft implantation in the left EIA

**Fig. 3:**
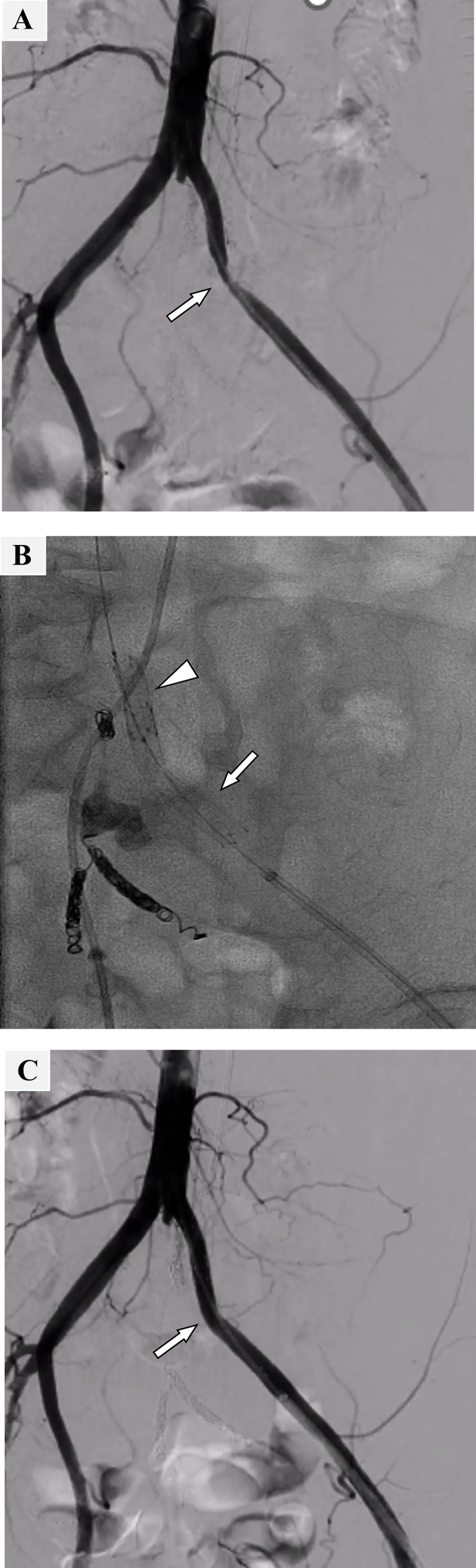
Stent graft placement in the left external iliac artery. (A) Digital subtraction angiography before stent graft placement. Stenosis was revealed in the left external iliac artery (EIA) (arrow). (B) Viabahn (7-mm/5-cm) was placed on the stenotic area in the EIA (arrow), and Viabahn (7-mm/2.5-cm) was placed to cover the crossing point with a ureteral stent (arrowhead). (C) EIA stenosis disappeared after stent graft placement (arrow).

Nine days after the embolization of the left IIA pseudoaneurysm, stent graft implantation was performed in the left EIA. After confirming complete hemostasis, 2 antiplatelet agents (ie, aspirin [100 mg] and clopidogrel [300 mg]) were orally loaded before the procedure. The stent graft was placed through a 7-Fr guiding sheath (Destination, Terumo, Tokyo, Japan) via the left common femoral artery. Subsequently, a 7-mm/5-cm Viabahn (W.L. Gore Associates, Flagstaff, AZ) was implanted in the stenotic area in the left EIA. Furthermore, a 7-mm/2.5-cm Viabahn was implanted to cover the crossing point of the left EIA and D-J stent to prevent future vascular injury associated with the contact of the ureteral stent (Fig. 3).

The patient was discharged 2 days after stent graft placement without any complications. Dual antiplatelet therapy (DAPT) (ie, aspirin [100 mg] daily and clopidogrel [75 mg] daily) was administered after the procedure. To date, 10 months after the endovascular treatment, no hemorrhagic complications have occurred, and the stent graft in the left EIA has remained patent. The patient is receiving chemotherapy for a recurrent lesion showing gradual enlargement.

## Discussion

URAF is an extremely rare condition in which the ureter and rectum simultaneously form a fistula with an artery, and it has previously been described in only a few case reports [[2], [3], [4]]. To the best of our knowledge, this is the first report in which URAF with EIA stenosis was successfully treated using an endovascular approach.

UAF is categorized into 2 types: primary UAF, which is caused by an abnormality of the artery (e.g., aneurysm and vascular malformation) and secondary UAF, which is caused by invasive treatments in the pelvis [5]. Surgery and radiation therapy in the pelvis and ureteral stent placement are considered risk factors for secondary UAF. Regarding AEF, although no coherent reports of risk factors are available, it is mainly caused by vascular abnormalities, including aneurysms and vascular malformations, and postoperative complications [6,7]. In our patient, it was suggested that fistula formation between the weakened arterial wall and surrounding organs was provoked by necrosis of a large recurrent tumor invading the ureter and rectum.

In the case of UAF that only involved the IIA, embolization of the affected artery is safely conducted [8]. However, in the case of AEF, surgical treatment is required in most cases because the risk of infection and coil migration into the bowel is high and endovascular treatment alone is rarely successful in the case of bleeding. In this case, the patient also had a rectal-arterial fistula, and similar risks were of concern. However, endovascular treatment was preferred because of the patient’s unstable condition and severe adhesions in the pelvis resulting from previous surgery and radiotherapy, which made the surgical approach unfeasible. Furthermore, because ileostomy was created, the risk of infection was expected to be low. Embolization was performed using coils and the NBCA mixture. Because the pelvic arterial system has a rich vascular network [9,10], the affected left IIA forming a pseudoaneurysm was embolized with the NBCA mixture to occlude small vessels that could potentially serve as collateral pathways. Furthermore, because of the patient’s shock status and coagulopathy, the procedure was performed urgently. These clinical factors necessitated the use of the NBCA mixture to achieve effective embolization.

In our patient, the left EIA was stenosed due to tumor invasion and prior radiation therapy. A stent graft was placed to prevent the risk of future bleeding and occlusion. Reports addressing the treatment of EIA stenosis secondary to tumor invasion are limited [11,12]. Treatment strategies, including stent graft placement and bypass surgery, are determined based on the underlying pathology and the patient’s condition. DAPT is generally recommended for at least 6 months following treatment of femoral arteriosclerosis with Viabahn [13,14]; however, the optimal course of antiplatelet therapy for arterial stenosis induced by tumor invasion remains unclear. Furthermore, our patient had a high risk of rebleeding. Therefore, the antiplatelet therapy regimen should be tailored according to the patient’s clinical course. In our patient, we intended to administer DAPT for >6 months, with dose adjustments considered if bleeding complications occurred. Arterial stenosis caused by tumor invasion carries the risk not only of thrombotic occlusion of the stent but also of occlusion due to tumor overgrowth, necessitating long-term follow-up with contrast-enhanced CT or ultrasonography.

Although this patient presented with life-threatening URAF with an unstable clinical condition, transarterial embolization was successfully achieved. Furthermore, stent graft placement in the EIA prevented the risk of future hemorrhage and occlusion. Endovascular treatment is less invasive compared to surgical intervention and can be a valuable option for patients in poor general condition who are not eligible for surgery. Furthermore, it demonstrates clinical utility in cases where surgical approaches are challenging due to postoperative or postradiation adhesions, or tumor invasion. Endovascular treatment is considered essential for managing URAF in hemodynamically unstable patients and arterial stenosis due to tumor invasion in patients with advanced-stage cancer.

## Patient consent

We certify that we have obtained all appropriate patient consent forms.

In the form the patient has given her consent for her images and other clinical information to be reported in the journal. The patient understand that her name and initial will not be published and due efforts will be made to conceal her identity.
